# Baricitinib and Infliximab Mitigate the Endothelial-to-Mesenchymal Transition (EndMT) Induced by Cytokines in HUVECs

**DOI:** 10.3390/ijms26178672

**Published:** 2025-09-05

**Authors:** Amelia Barilli, Rossana Visigalli, Giulia Recchia Luciani, Eleonora Crescini, Valeria Dall’Asta, Bianca Maria Rotoli

**Affiliations:** Laboratory of General Pathology, Department of Medicine and Surgery, University of Parma, 43125 Parma, Italy; amelia.barilli@unipr.it (A.B.); rossana.visigalli@unipr.it (R.V.); giulia.recchialuciani@unipr.it (G.R.L.); eleonora.crescini@studenti.unipr.it (E.C.); biancamaria.rotoli@unipr.it (B.M.R.)

**Keywords:** COVID-19, baricitinib, EndMT, endothelium, IFNγ, infliximab, macrophages, TNFα, TGFβ

## Abstract

Endothelial-to-mesenchymal transition (EndMT) is associated with various pathologies including cardiovascular, inflammatory, and fibrotic diseases or neoplasia. Concerning COVID-19, multiple organ dysfunctions and long COVID syndrome are mediated by microvascular damage and, recently, the role of SARS-CoV-2 peptide fragments in the induction of EndMT was demonstrated. Here, we investigated the immune-mediated effects of Spike S1 of SARS-CoV-2 on EndMT and demonstrated that cytokines secreted by S1-activated macrophages, mainly TNFα + IFNγ, also induce the phenotypical switch in HUVECs. In particular, a loss of the typical cobblestone morphology is observed, along with a huge reduction in endothelial adhesion molecules, such as vWF, CD31, and VE-cadherin, and a concomitant acquisition of mesenchymal markers, such as N-cadherin and FSP1 protein. In addition, the combined use of the drug infliximab, targeting TNFα, and baricitinib, an inhibitor of the JAK-STAT pathway, hinders the phenotypical changes by restoring the proper expression of endothelial markers. The protective effect of these drugs is evident not only when they are added to the culture medium together with the trigger, but also when added later, i.e., once EndMT has been started. These findings reinforce the role of COVID-19-associated cytokine storm in endothelial dysfunction and in the onset of the fibrotic process and sustain the clinical relevance of infliximab and baricitinib for the prevention of vascular damage.

## 1. Introduction

Currently, the overall severity and mortality of COVID-19 are greatly diminished compared to earlier stages of the pandemic, thanks to vaccines, effective therapies, and less severe variants.

However, COVID-19 can still cause severe illness, including long-term complications, that remain a serious public health concern. This post-COVID-19 condition is termed “long COVID”, “post-acute sequelae of COVID-19”, or “post-COVID-19 syndrome”, and manifests with a wide range of symptoms, including neurological, gastrointestinal, pulmonary, and cardiovascular complications [1,2,3,4].

The pathophysiological mechanisms underlying long COVID-19 remain unclear. Current hypotheses suggest that viral persistence and immune dysfunction play central roles; the resulting consequences include cytokine storm, organ damage, chronic inflammation, and an altered immune status, which collectively contribute to the prolonged symptoms observed under this condition [5]. In this context, the overexpression of proinflammatory cytokines, such as interleukin-6 (IL-6), tumor necrosis factor-alpha (TNFα), interleukin-1β (IL-1β), and interferon-gamma (IFNγ) [6,7], plays a central pathogenetic role. In particular, IFNγ and canonical NF-κB signaling, associated with TNFα, appear to be the most prevalent signaling pathways [5].

On the other hand, cytokines are also responsible for endothelial activation and damage, which likely accounts for the persistent endothelial dysfunction underlying the non-respiratory symptoms of long COVID [8,9]. Moreover, cytokines can trigger and drive endothelial-to-mesenchymal transition (EndMT), a phenomenon by which endothelial cells (ECs) progressively lose typical endothelial features and acquire mesenchymal fibroblast-like characteristics, transitioning from a differentiated to an undifferentiated state [10,11]. During this process, cells lose endothelial cell-specific proteins such as CD31/PECAM, von Willebrand factor (vWF), and vascular–endothelial cadherin (VE-cadherin) and initiate the expression of mesenchymal markers including α-smooth muscle actin (α-SMA), neural cadherin (N-cadherin), fibroblast-specific protein 1 (FSP1), fibronectin, vimentin, and type I and type III collagen (ColI/III). Among cytokines, transforming growth factor-beta (TGF-β) is notably considered the main EndMT inducer [12,13,14]. However, EndMT is a complex biological process that is modulated by several redundant TGF-β-dependent and non-dependent mechanisms related to the specific cellular-status context [15]. Actually, inflammatory mediators, such as IL-1β, TNF-α, and nuclear factor kappa B (NF-κB) transcription factor, can activate endothelial cells and promote the EndMT process [16,17,18].

Recently, the study by Baldassarro et al. explored the possible impact of SARS-CoV-2-induced endothelial-to-mesenchymal transition (EndMT) on fibrosis, corroborating the hypothesis that the observed multiple organ dysfunctions and long COVID syndrome are mediated by microvascular damage [19].

In previous contributions, we recently addressed the effects of the inflammatory mediators released by Spike S1-treated macrophages on endothelial cells and demonstrated an impairment of cell proliferation and viability, along with an imbalance of eNOS/arginase metabolism [20]. In the current study, we explored whether the cytokines secreted by Spike-activated macrophages direct endothelial cells towards an endothelial-to-mesenchymal transition (EndMT). The underlying mechanisms were also investigated, along with the protective effect mediated by the drugs baricitinib and infliximab.

## 2. Results

**Figure 1:**
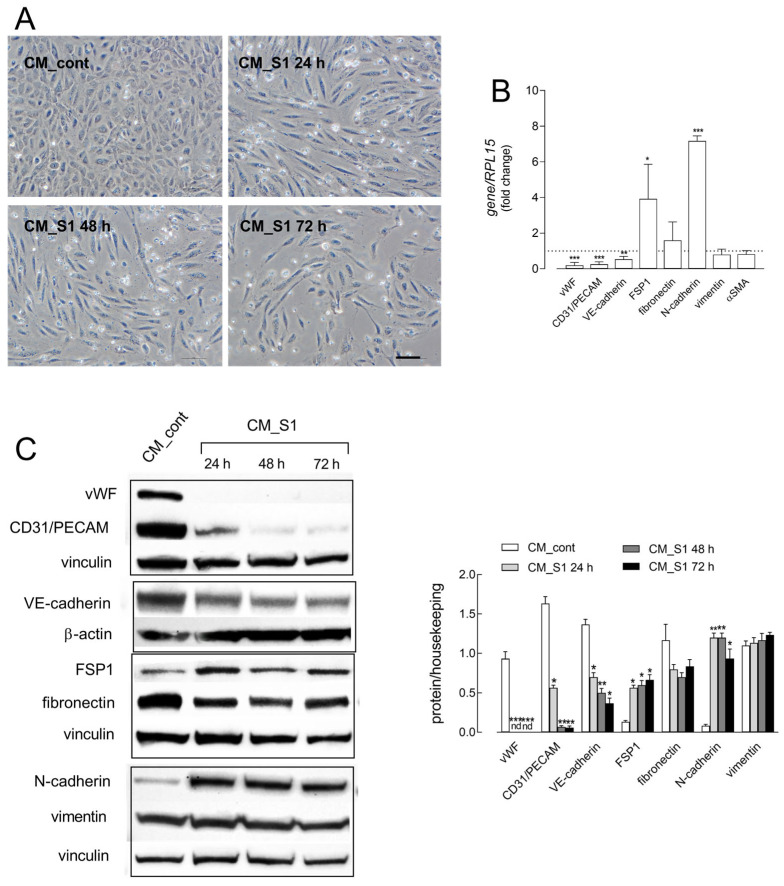
HUVECs were incubated in conditioned medium (CM) obtained from monocyte-derived macrophages, untreated (CM_cont) or treated with 5 nM S1 (CM_S1). (**A**) Phase-contrast microscopy images of cells treated for the indicated times. Bar = 10 µm. (**B**) After 24 h, the mRNA levels of the genes indicated were assessed with RT-qPCR and shown as fold change in CM_cont (=1). Bars are means ± SEM of three experiments performed in duplicate. * *p* < 0.05, ** *p* < 0.01, *** *p* < 0.001 vs. control with Student’s *t* test. (**C**) At the times indicated, protein expression was determined with Western blot, as detailed in Methods. Representative blots are shown (**left**), along with mean ± SEM of the densitometry analysis in three different experiments (**right**). * *p* < 0.05, ** *p* < 0.01, *** *p* < 0.001 vs. CM_cont with One-way ANOVA.

In order to explore the possible relationship between macrophage activation and EndMT in COVID-19, endothelial cells were incubated with conditioned medium obtained from Spike S1-treated macrophages (CM_S1) and the profile of endothelial and mesenchymal markers associated with EndMT was evaluated. First, as shown in Figure 1A, HUVEC exposure to CM_S1 already induces profound changes in morphology after 24 h of treatment, when cells lose their typical cobblestone shape and become elongated. This phenomenon is even more evident after 48 and 72 h of treatment, when a clear decrease in cell growth is also appreciable. In parallel, the molecular analysis performed under the same conditions highlights an evident loss of vasculature-specific markers and a concomitant increase in mesenchymal gene expression. Data in Figure 1B demonstrate, indeed, that CM_S1 causes a marked decrease in von Willebrand factor (vWF), platelet–endothelial cell adhesion molecule-1 (PECAM1/CD31), and vascular–endothelial cadherin (VE-cadherin/CDH5), as well as a significant increase in neural cadherin (N-cadherin) and fibroblast-specific protein-1 (FSP-1). Conversely, no change is observed in the expression of the other specific fibroblastic markers α-smooth muscle actin (α-SMA) and vimentin. At the protein level (Figure 1C), the total disappearance of vWF is observed already after 24 h of incubation; similarly, CD31/PECAM, already lowered after 24 h, also progressively disappears at longer times. The expression of VE-cadherin undergoes a more modest but still significant reduction. N-cadherin and FSP1 proteins also appear to be modulated coherently with mRNA levels, with a clear-cut induction of N-cadherin and a modest increase in FSP1; the lack of change in vimentin and fibronectin expression is confirmed also at the protein level. The morphological changes observed, along with the modulation of endothelial/mesenchymal markers, suggests that, under our experimental conditions, HUVECs undergo a transition from an endothelial to a mesenchymal phenotype.

**Figure 2:**
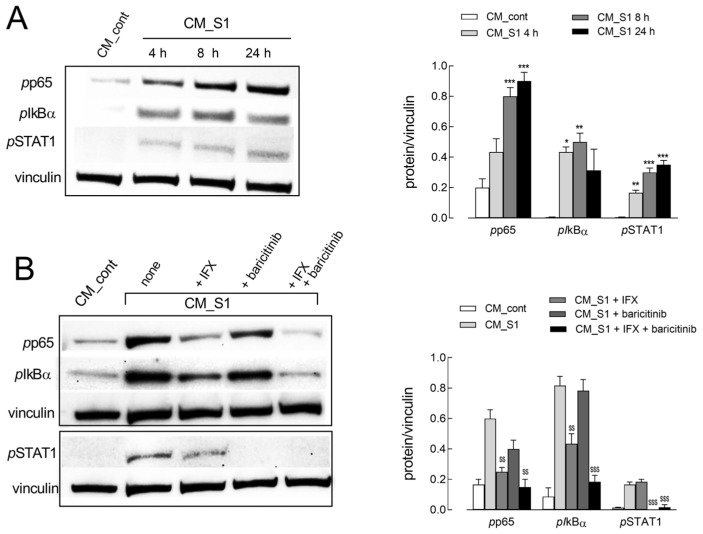
HUVECs were incubated in CM_cont or in CM_S1 in the absence (**A**) or in the presence of 100 µg/mL IFX or 1 µM baricitinib. At the times indicated (**A**) or after 8 h (**B**), the expression of the indicated proteins was determined with Western blot, as detailed in Methods. Representative blots are shown (**left**), along with mean ± SEM of the densitometry analysis in three different experiments (**right**). * *p* < 0.05, ** *p* < 0.01, *** *p* < 0.001 vs. CM_cont; ^$$^ *p* < 0.01, ^$$$^ *p* < 0.001 vs. CM_S1 with One-way ANOVA.

To investigate the molecular mechanisms underlying this phenotypic switch, the activation status of NF-κB and JAK/STAT signaling pathways, typically activated in acute inflammation and involved in the exacerbation of host inflammatory responses in COVID-19 patients [21], was then assessed. As shown in Figure 2A, CM_S1 treatment progressively and markedly induces the phosphorylation of both p65 and IkBα, the subunit and inhibitor of NF-κB, respectively, as well as of STAT1, confirming the activation of both transcription factors. Consistently, the addition of the drug baricitinib, the inhibitor of JAK/STAT, selectively suppresses STAT1 phosphorylation, while infliximab (IFX), a monoclonal anti-TNFα antibody, limits NF-κB signaling by completely preventing p65 activation and significantly reducing the phosphorylation of IkBα. The combined treatment with the two drugs fully hinders the CM_S1-dependent activation of both inflammatory pathways (Figure 2B).

**Figure 3:**
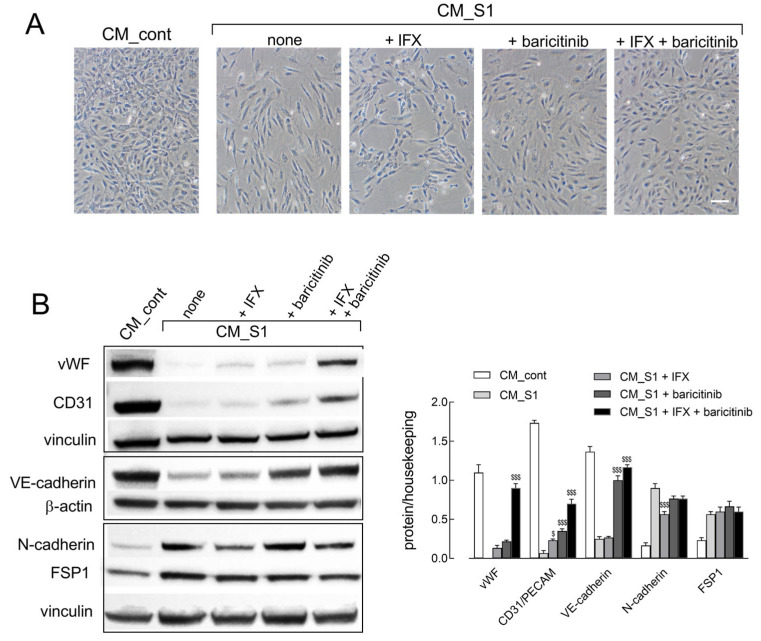
HUVECs were incubated for 72 h in CM_cont or in CM_S1, in the absence or in the presence of 100 µg/mL IFX or 1 µM baricitinib. (**A**) Phase-contrast microscopy images of cells incubated under the indicated conditions. Bar = 10 µm. (**B**) The expression of the indicated proteins was determined with Western blot, as detailed in Methods. Representative blots are shown (**left**), along with mean ± SEM of the densitometry analysis (**right**) in three different experiments. ^$^ *p* < 0.05, ^$$$^ *p* < 0.001 vs. CM_S1 with One-way ANOVA.

In line with these findings, the addition of either IFX or baricitinib to CM_S1-treated cells only partially restores the typical morphology of endothelial cells; conversely, a much more evident preservation of the polygonal morphology is observed when the two drugs are simultaneously added (Figure 3A). Under the same experimental condition, the pattern of expression of endothelial and mesenchymal markers also confirms the protective effects of IFX and baricitinib on EndMT induction (Figure 3B). Indeed, the CM_S1-dependent disappearance of CD31 is partially restored by the combination of IFX + baricitinib, while the loss of VE-cadherin, still evident in the presence of IFX, is prevented by baricitinib. The latter is also effective in preventing the induction of N-cadherin, which is, instead, insensible to IFX. Only the CM_S1-mediated induction of FSP1 protein is unaffected by any drug. Thus, our findings suggest that these drugs, employed together, can at least partially prevent EndMT and preserve the endothelial phenotype in CM_S1-treated cells.

**Figure 4:**
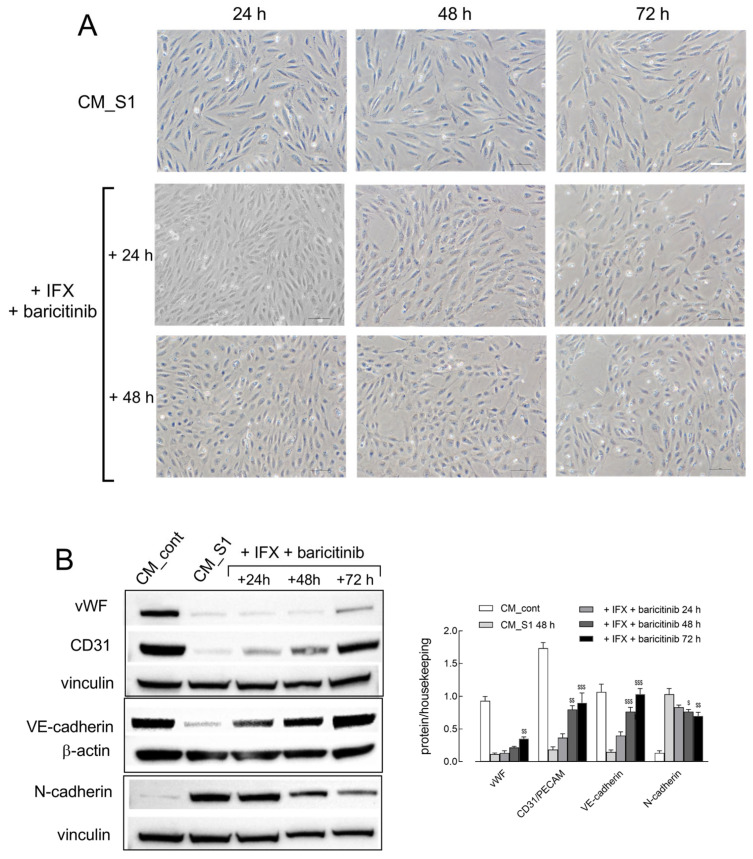
(**A**) HUVECs, treated with CM_S1 for 24, 48, or 72 h, were then further added to 100 µg/mL IFX + 1 µM baricitinib. After 24 h and 48 h, phase-contrast microscopy images of representative fields were acquired. Bar = 10 µm. (**B**) HUVECs were treated for 48 h with CM_S1 and, after this time, the two drugs were added to the cultures. The expression of the indicated proteins was determined with Western blot at the indicated times, as detailed in Methods. Representative blots are shown, along with mean ± SEM of the densitometry analysis (**right**) in three different experiments. ^$^ *p* < 0.05, ^$$^ *p* < 0.01, ^$$$^ *p* < 0.001 vs. CM_S1 with One-way ANOVA.

Interestingly, the same protective effect is also observed when the drugs are added to the culture medium once EndMT has already been started. To address this issue, HUVECs have been incubated in CM_S1 for 24, 48, or 72 h; then, IFX + baricitinib were added for an additional 24 and 48 h and the images of cells under the different experimental conditions were acquired. As shown in Figure 4A, IFX + baricitinib completely reverted the transition when added after 24 h treatment with CM_S1; indeed, a complete restoration of the typical cobblestone morphology was observed after 48 h in the presence of the drugs. The curative effect of IFX + baricitinib was also evident, albeit less pronounced, when the two inhibitors were added to HUVECs maintained in CM_S1 for 48 h or 72 h. Under this condition, i.e., upon addition of the drugs to cells treated with CM_S1 for 48 h, the pattern of expression of EndMT markers was assessed over time (Figure 4B). Results obtained show that after 72 h, VE-cadherin and CD31 are totally restored by the addition of IFX + baricitinib, while the increase in N-cadherin declines. The recovery of vWF protein is also observed, although less pronounced.

**Figure 5:**
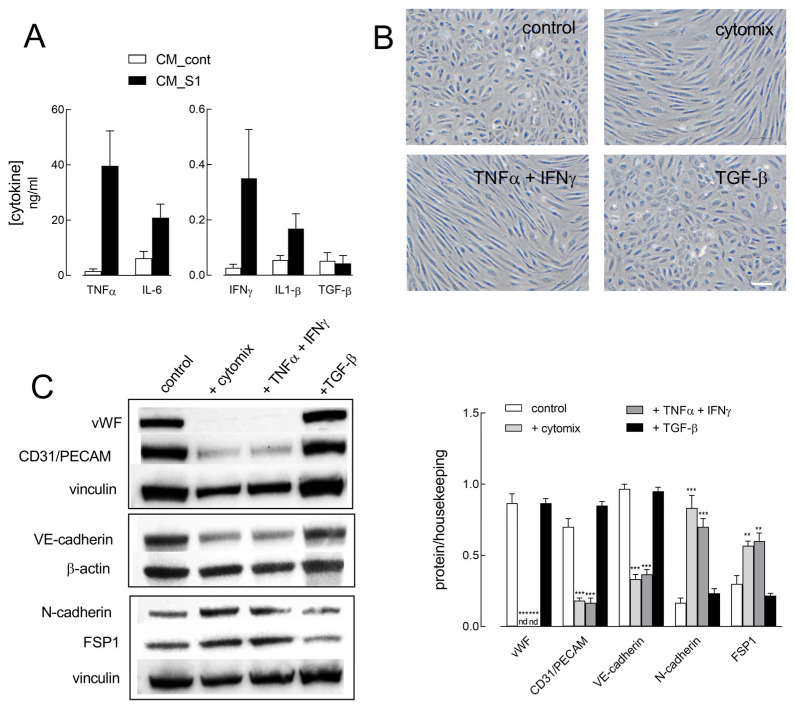
(**A**) The amount of the indicated cytokines in CM_cont and CM_S1 from six different donors was determined as described in Methods. (**B**,**C**). HUVECs were incubated for 72 h in RPMI1640 medium, in the absence (control) or in the presence of cytomix (TNFα + IL-6 + IFNγ + IL-1β), or with TNFα + IFNγ or with TGF-β alone. (**B**) Phase-contrast microscopy images of cells incubated in the indicated media. Bar = 10 µm. (**C**) The expression of the indicated proteins was determined with Western blot, as detailed in Methods. Representative blots are shown (**left**); data of densitometry analysis are the mean ± SEM of three different experiments (**right**; nd, not detectable). ** *p* < 0.01, *** *p* < 0.001 vs. control with One-way ANOVA.

In an effort to elucidate the cellular mechanisms responsible for the observed events, we next addressed the effects of inflammatory cytokines, expected to be secreted by macrophages in the incubation medium upon exposure to the Spike S1 protein, namely TNFα, IL-6, IFNγ, IL-1β, and TGF-β. As shown in Figure 5A, higher amounts of both TNFα and IL-6 are present in CM_S1 than in CM_cont (i.e., in the conditioned medium of untreated macrophages); IFNγ and IL-1β levels are also increased in CM_S1, although at lower concentrations than TNFα and IL-6. On the contrary, the secretion of TGF-β, which is very low in CM_cont, is not modified in CM_S1. Hence, we exposed HUVECs to these cytokines and addressed their effect on cell morphology and on the modulation of endothelial and mesenchymal marker proteins. In particular, cells were treated for 72 h with a mix of the four cytokines TNFα, IL-6, IFNγ, and IL-1β (hereafter named “cytomix”) with TNFα + IFNγ or with TGF-β alone, widely recognized as an inducer of EndMT. As shown in Figure 5B, only cytomix and TNFα + IFNγ are able to induce an elongated shape in endothelial cells, as already described in a previous contribution [22]; conversely, no gross change in endothelial morphology is evident upon incubation with TGF-β (Figure 5A). Similarly, cytomix and TNFα + IFNγ, but not TGF-β, causes the loss of endothelial proteins vWF, CD31, and VE-cadherin, along with a concomitant increase in N-cadherin and FSP1 markers, ascribing these cytokines a role as triggers of the changes induced in HUVECs by CM_S1.

## 3. Discussion

A growing body of evidence has demonstrated that EndMT is associated with various pathologies, including cardiovascular, inflammatory, and fibrotic diseases, or neoplasia. As far as COVID-19 is concerned, the study by Baldassarro et al. has recently demonstrated that SARS-CoV-2 peptide fragments induce EndMT in primary microvascular endothelial cells, corroborating the hypothesis of the role of SARS-CoV-2-mediated microvascular damage in the onset of the multiple organ dysfunctions typical of the disease and of long COVID syndrome [19]. Here, by further investigating the immune-mediated effects of Spike S1 from SARS-CoV-2, we demonstrate that cytokines secreted by S1-activated macrophages also induce EndMT in endothelial cells in vitro, as evidenced by the loss of endothelial adhesion molecules and the concomitant acquisition of mesenchymal markers. In addition, we also demonstrate that the drugs infliximab (IFX) and baricitinib hinder the phenotypical changes by restoring the typical cobblestone morphology and the proper expression of endothelial markers.

The direct role of immune cells in EndMT induction has been evaluated only in a few studies, where co-cultures of endothelial cells and macrophages were employed, or where, alternatively, endothelial cells were exposed to supernatants of activated macrophages [23,24,25]; in those studies, cytokines secreted by immune cells emerged as the main trigger for EndMT in cardiovascular diseases. According to our results, it is reasonable to assume that the induction of EndMT in COVID-19 is also ascribable to the presence of several cytokines secreted by macrophages in CM_S1 medium, including IL-1β, IL-6, TNFα, and IFNγ.

Among the variety of EndMT inducers, these four cytokines have been described as main initiators of EndMT in different models of endothelial cells [15,18]. By addressing their effects, we conclude here that TNFα and IFNγ are specifically involved in triggering a phenotypical switch in HUVECs. Indeed, the exposure to TNFα + IFNγ determines a complete loss of vWF and a reduction in CD31 and VE-cadherin, along with a concomitant up-regulation of N-cadherin and FSP1. Conversely, neither vimentin nor fibronectin expression were modified under our experimental conditions, while α-SMA, not even detectable, was only expressed in cells treated with 5 µM bleomycin, a known inducer of EndMT (see Figure S1). This pattern of expression of EndMT markers, comparable in CM_S1- and TNF + IFNγ-treated cells, supports the hypothesis that a partial transition, rather than a complete phenotypic shift, occurs under our experimental conditions, in which intermediary cells still co-express endothelial and mesenchymal markers. Moreover, while in complete EndMT endothelial cells show enhanced proliferative and migratory capacity [15,26], we have previously described a cytokine-mediated proliferative decline of HUVECs under our experimental conditions [22].

Other unexpected findings concern TGF-β, a widely recognized driver of complete EndMT. Indeed, in our hands, the treatment of human macrophages with Spike S1 does not cause any increase in TGF-β secretion; moreover, the same cytokine does not induce any sign of EndMT in HUVECs, at least up to 72 h. A similar result has been obtained in the same endothelial model by other groups [27,28]. Möbus et al., in particular, show that TGF-β’s effects on EndMT induction in HUVECs are limited and transient, and, hence, suggest that its pro-fibrotic action may require another initial trigger or an interplay with other cells; actually, the same study identifies bleomycin, and not TGF-β, as an agent able to induce a rapid and extensive shift in the EndMT transcriptional program in HUVECs.

At the molecular level, the EndMT program is orchestrated by a variety of biochemical, biomechanical, and environmental signals leading to the activation of various signaling pathways that result in the activation of transcription factors, primarily NF-κB [15]. Here, we show that both NF-κB and JAK-STAT act as key drivers of Spike-induced EndMT in HUVECs; indeed, the inhibition of these signaling pathways by the drug infliximab that blocks TNFα, and baricitinib, inhibitor of JAK-STAT, is able to mitigate EndMT.

The correlation between IFX and baricitinib and EndMT has not been properly documented thus far. As far as infliximab is concerned, this TNFα antagonist has been previously reported to improve endothelial dysfunction in different diseases. First, it is widely used in clinical practice for patients with Crohn’s disease [29], and to improve microvascular endothelial dysfunction in rheumatoid arthritis [30]; moreover, administration of IFX in patients with active AASV (anti-neutrophil cytoplasmic antibody-associated systemic vasculitis) reduces systemic inflammation and improves endothelial function [31]. The same drug has also proven able to mitigate the arterial wall inflammation in patients with Kawasaki Disease [32], and, in the same disease, to induce, with intravenous immunoglobulin and atorvastatin, a beneficial transition in gene expression patterns concerning inflammation and EndMT [33]. Baricitinib, included among the JAKinibs used in the therapy for rheumatoid arthritis [34], also emerged as a promising therapy for the treatment of skin fibrosis in Diffuse Cutaneous Systemic Sclerosis and Digital Ulcers [35]. In COVID-19, baricitinib has been recognized as one of the first-choice immunomodulators for the treatment of severe patients [36,37]. We previously demonstrated that this drug mitigates the inflammatory response induced in immune and endothelial cells by the Spike S1 protein in vitro [38] and, more recently, that, together with IFX, it corrects the CM_S1-dependent eNOS/arginase imbalance [20]. Consistently, we show here that, while IFX and baricitinib only modestly counteract EndMT when employed alone, they almost completely hinder the phenotypical shift when combined, preventing the modulation of endothelial and mesenchymal markers by CM_S1, and restoring the original cobblestone-like morphology. Interestingly, the ability to counteract EndMT and to restore the endothelial phenotype is evident both when the two drugs are administered together with CM_S1 and when added later, once the process is started. This finding deserves particular attention, as it also suggests a curative effect of the combined use of IFX + baricitinib for ongoing EndMT.

In conclusion, our data reinforce the importance of cytokine storm in causing vascular damage in COVID-19 and support the clinical relevance of the combined use of the two drugs to mitigate long COVID-associated EndMT and fibrosis.

## 4. Materials and Methods

### 4.1. Cell Culture and Experimental Treatments

Human monocyte-derived macrophages were obtained as already described from monocytes isolated from the buffy coats of anonymous healthy donors [39]. The study protocol was approved by the local ethics committee (460/2021/TESS/UNIPR). For the experiments, macrophages were incubated with or without 5 nM of the S1 subunit of the SARS-CoV-2 Spike recombinant protein (ARG70218; Arigo Biolaboratories, Taiwan, China). Conditioned media from six different donors were collected after 24 h from control (CM_cont) and S1-treated MDMs (CM_S1) and pooled for further use.

Human Umbilical Vein Endothelial Cells (HUVECs) were purchased from Thermo Fisher Scientific (Monza, Italy). Cells were cultured in Human Large Vessel Endothelial Basal Medium added with Large Vessel Endothelial Supplement (LVES) and used between 1 and 6 passages. For experiments, 5 × 10^4^ cells/mL were seeded in multi-well plates and treated according to the experimental design. When HUVECs were incubated with CM_cont or CM_S1, LVES was added to the medium to provide the supplements required for endothelial cell growth, lacking in RPMI1640. When required, infliximab (IFX, 200 µg/mL) or baricitinib (1 µM) were added to CM_S1. In experiments evaluating the effects of cytokines, HUVECs were incubated in RPMI1640 supplemented with LVES; 5 ng/mL of IFNγ or IL-1β, or 50 ng/mL of TNFα or IL-6 (R&D Systems by Bio-Techne, Milano, Italy) were added according to the experimental plan. TGF-β (20 ng/mL; R&D Systems) was employed as a known inducer of EndMT.

### 4.2. RT-qPCR Analysis

For gene expression analysis, cells, seeded in 24-well plates, were treated according to the experimental design and total RNA was analyzed as already described [39]. Forward and reverse primer pairs employed are listed in Table 1. Results are shown as fold change relative to the control group (=1), calculated with the ∆∆Ct method.

### 4.3. Western Blot Analysis

For the analysis of protein expression, cell lysates were prepared using LDS sample buffer (Thermo Fisher Scientific) and analyzed as already described [40]. The following antibodies (1:2000, Cell Signaling Technology, Euroclone, Pero-Milano, Italy) were employed: rabbit polyclonal anti-VWF; anti-VE-Cadherin; anti-N-cadherin; anti-phospho-STAT1 (Tyr705); anti-phospho-NF-κB p65 (Ser536); mouse monoclonal anti-phospho-IκBα (Ser32/36); rabbit polyclonal anti-FSP1 (1:4000 Thermo Fisher). Anti-vinculin mouse monoclonal antibody (1:2000, Merck, Milano, Italy), or anti-β-Actin (1:3000 Cell Signaling), were employed as loading controls. Western blot images were captured using an iBright FL1500 Imaging System (Thermo Fisher Scientific) and analyzed with iBright Analysis Software (version 5.0).

### 4.4. Cytokine Analysis

The amounts of TNFα, IL-1β, IL-6, IFNγ, and TGF-β secreted in the culture medium of the control (CM_cont) and Spike S1-activated macrophages (CM_S1) were measured with specific Human Quantikine ELISA kits (R&D Systems, Bio-Techne, Milano, Italy).

### 4.5. Statistical Analysis

Statistical analyses were performed using GraphPad Prism 9 (GraphPad Software, Boston, MA, USA). *P*-values were determined using Ordinary One-way ANOVA (with post hoc Dunnet’s multiple comparisons test) or Student’s *t*-test, as indicated in the legend of each figure. A *p*-value of less than 0.05 was considered indicative of statistical significance.

### 4.6. Materials

Endotoxin-free fetal bovine serum was sourced from Thermo Fisher Scientific. The anti-TNFα monoclonal antibody infliximab, IFX (Remsima), was from Celltrion Healthcare Italy (Milano, Italy), while baricitinib was from Vinci-Biochem. All other chemicals were purchased from Merck (Milano, Italy), unless stated otherwise.

## Figures and Tables

**Table 1:** Sequences of the primer pairs employed for RT-qPCR analysis.

Gene/Protein Name	Forward Primer	Reverse Primer
*VWF/von Willebrand factor*	TGGAGGGAGGAGAGATTGAG	CCCAGCAGCAGAATGATGTA
*PECAM/CD31*	AACAGTGTTGACATGAAGAGCC	TGTAAAACAGCACGTCATCCTT
*CDH5/VE-CAD*	AAGCGTGAGTCGCAAGAATG	TCTCCAGGTTTTCGCCAGTG
*S100A4/FSP1*	GATGAGCAACTTGGACAGCAA	CTGGGCTGCTTATCTGGGAAG
*CDH2/N-CAD*	TCAGGCGTCTGTAGAGGCTT	ATGCACATCCTTCGATAAGACTG
*FN-1/fibronectin*	GAGAATAAGCTGTACCATCGCAA	CGACCACATAGGAAGTCCCAG
*VIM/vimentin*	TGCCGTTGAAGCTGCTAACTA	CCAGAGGGAGTGAATCCAGATTA
*ACTA1/αSMA*	GCTGTTTTCCCATCCATTGT	TTTGCTCTGTGCTTCGTCAC
*RPL15/RPL15*	GCAGCCATCAGGTAAGCCAAG	AGCGGACCCTCAGAAGAAAGC

## Data Availability

Data are available at https://osf.io/tcwh5/files/osfstorage/6880e546e3bc3992dc84fb07, accessed on 29 August 2025.

## Supplementary Materials

The following supporting information can be downloaded at: https://www.mdpi.com/article/10.3390/ijms26178672/s1.
