# Supplementary material for: Baricitinib and Infliximab Mitigate the Endothelial-to-Mesenchymal Transition (EndMT) Induced by Cytokines in HUVECs
Source: Int J Mol Sci. 2025 Sep 5;26(17):8672. doi: 10.3390/ijms26178672 (PMC12429012; doi:10.3390/ijms26178672)
Supplement: Supplementary file 1 [file ijms-26-08672-s001.zip › ijms-3809551-supplementary.pdf]

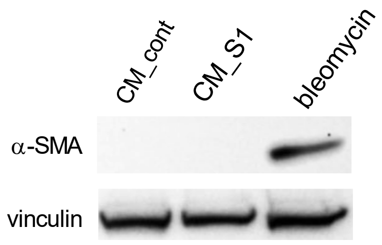

Figure 1S. HUVECs were incubated for 72 h in conditioned medium (CM) obtained from monocytes-derived macrophages, either untreated (CM_cont) or treated with 5 nM S1 (CM_S1), or with 5 μM bleomycin. At the end, the expression of α-SMA was determined with Western Blot analysis, as detailed in Methods.
